# Substance P Causes Seizures in Neurocysticercosis

**DOI:** 10.1371/journal.ppat.1002489

**Published:** 2012-02-09

**Authors:** Prema Robinson, Armandina Garza, Joel Weinstock, Jose A. Serpa, Jerry Clay Goodman, Kristian T. Eckols, Bahrom Firozgary, David J. Tweardy

**Affiliations:** 1 Department of Medicine, Baylor College of Medicine, Houston, Texas, United States of America; 2 Division of Gastroenterology, Tufts New England Medical Center, Boston, Massachusetts, United States of America; 3 Department of Pathology, Baylor College of Medicine, Houston, Texas, United States of America; McGill University, Canada

## Abstract

Neurocysticercosis (NCC), a helminth infection of the brain, is a major cause of seizures. The mediators responsible for seizures in NCC are unknown, and their management remains controversial. Substance P (SP) is a neuropeptide produced by neurons, endothelial cells and immunocytes. The current studies examined the hypothesis that SP mediates seizures in NCC. We demonstrated by immunostaining that 5 of 5 brain biopsies from NCC patients contained substance P (SP)-positive (+) cells adjacent to but not distant from degenerating worms; no SP+ cells were detected in uninfected brains. In a rodent model of NCC, seizures were induced after intrahippocampal injection of SP alone or after injection of extracts of cysticercosis granuloma obtained from infected wild type (WT), but not from infected SP precursor-deficient mice. Seizure activity correlated with SP levels within WT granuloma extracts and was prevented by intrahippocampal pre-injection of SP receptor antagonist. Furthermore, extracts of granulomas from WT mice caused seizures when injected into the hippocampus of WT mice, but not when injected into SP receptor (NK1R) deficient mice. These findings indicate that SP causes seizures in NCC, and, suggests that seizures in NCC in humans may be prevented and/or treated with SP-receptor antagonists.

## Author Summary

Neurocysticercosis (NCC), is a helminth infection of the brain that is caused by *Taenia solium*. NCC is the major cause of acquired seizures worldwide. Live *Taenia solium* parasites in the brain of NCC patients are surrounded by little or no inflammation. Seizures are thought to result not from parasitic infection per se, but from the chronic granulomatous host response initiated by dying cysts. Antiparasitic drugs can be used to kill the parasites, but the symptoms may worsen due to the host inflammatory responses being stimulated by the dying parasites. The mediators that are responsible for mediating seizures in NCC are not known; identification of the seizure mediator(s) may lead to prevention/treatment of seizures with specific antagonists. In this important study, we demonstrated that Substance P, a neuropeptide and pain transmitter is responsible for seizures in NCC. These studies have potential implications for treatment and prevention of seizures in the setting of NCC.

## Introduction

Neurocysticercosis (NCC) is a parasitic infection of the human central nervous system that is caused by the pig tapeworm *Taenia solium*. NCC is the major cause of acquired seizures worldwide and now accounts for up to 10% of emergency room visits for seizures in the southwestern United States [1]–[7].

Live *T. solium* cysts in the brain of NCC patients are surrounded by little or no inflammation. Seizures are thought to result from the granulomatous host response initiated by dead or dying cysts rather than mediators produced by the parasite itself [5], [8]–[11]. Since antihelmintic medications kill live cysts, a dilemma arises regarding treatment with these agents.

The mediators responsible for inducing seizures in NCC are not known; their identification may lead to more effective strategies for prevention and/or treatment of seizures in this disease. Substance P (SP) is a neuropeptide within the tachykinin family produced by neurons, endothelial cells and immunocytes such as lymphocytes and macrophages. Receptors for SP (NK1R) are expressed by cells within and outside the central nervous system including neurons, endothelial cells and immunocytes [12]–[16]. SP signaling is involved in nociception [17] and neuropathic inflammation. A few reports implicate SP in the pathogenesis of seizures including one in which SP amplified seizure responses in rats [18], [19].

The studies reported herein were undertaken to examine the hypothesis that SP is the epileptogenic agent in NCC. Our findings in brain biopsies of patients with NCC and in rodent models of NCC indicate that SP, produced within cysticercosis granulomas, causes seizures and suggest that seizures in NCC patients can be prevented and/or treated with SP receptor antagonists currently available in the clinic.

## Results

### SP-positive (+) cells are demonstrated in the brains of patients with NCC

**Figure 1:**
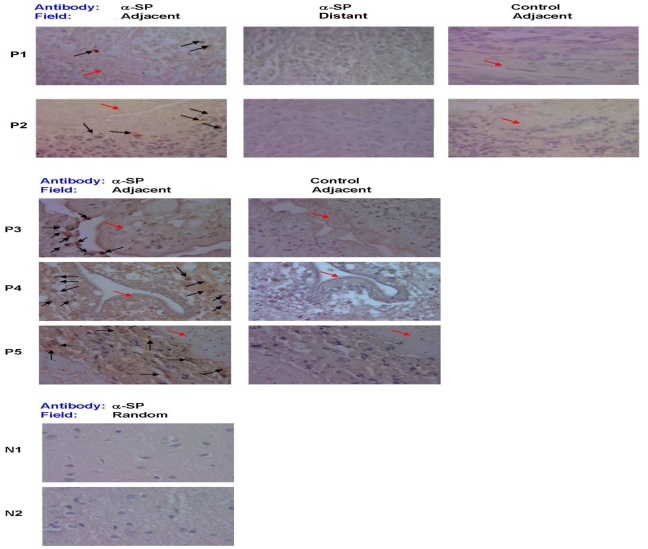
SP immunostaining of brain tissue samples from NCC-infected and uninfected patients. Photomicrographs of brain biopsy specimens of NCC-infected patients (P1 through P5) and brain autopsy specimens of NCC-uninfected patients (N1 and N2) are shown. Specimen slides were stained with specific polyclonal rabbit anti-SP antibody (α-SP) or control rabbit serum. Fields shown are either adjacent to the parasite (indicated by red arrow) or distant from parasite [when specimen size permitted (P1 and P2)], as indicated. SP+ cells are indicated by black arrows, 1000× magnification. Data shown is representative of one independent experiment.

To determine if SP-producing cells can be detected in the brains of NCC patients with seizures, particularly within the region of granulomatous inflammation surrounding the dead or dying cyst, we performed immunohistochemistry on brain tissue specimens from patients with and without NCC. Included were 5 patients with NCC who underwent craniotomy and brain biopsy to remove intraparenchymal cysts and 2 individuals who died from non-neurological causes (Figure 1). SP peptide expression was readily detected within cells adjacent to parasite remnants in each of the 5 NCC brain biopsy specimens; the level of expression scored an average of 2±0.71. In contrast, no SP+ cells were found distant from the parasite in the NCC biopsy specimens that included sufficient brain tissue; these distant areas scored 0.5±0 (p<.05, Mann-Whitney test). Autopsy specimens from patients without NCC displayed no SP+ staining cells (score = 0.5±0; p<0.05, Mann-Whitney test). These findings are consistent with the hypothesis that SP is produced within the granulomatous inflammation that surrounds dead or dying cysts in NCC.

### Injection of SP into the hippocampus of rats induces seizures

**Figure 2:**
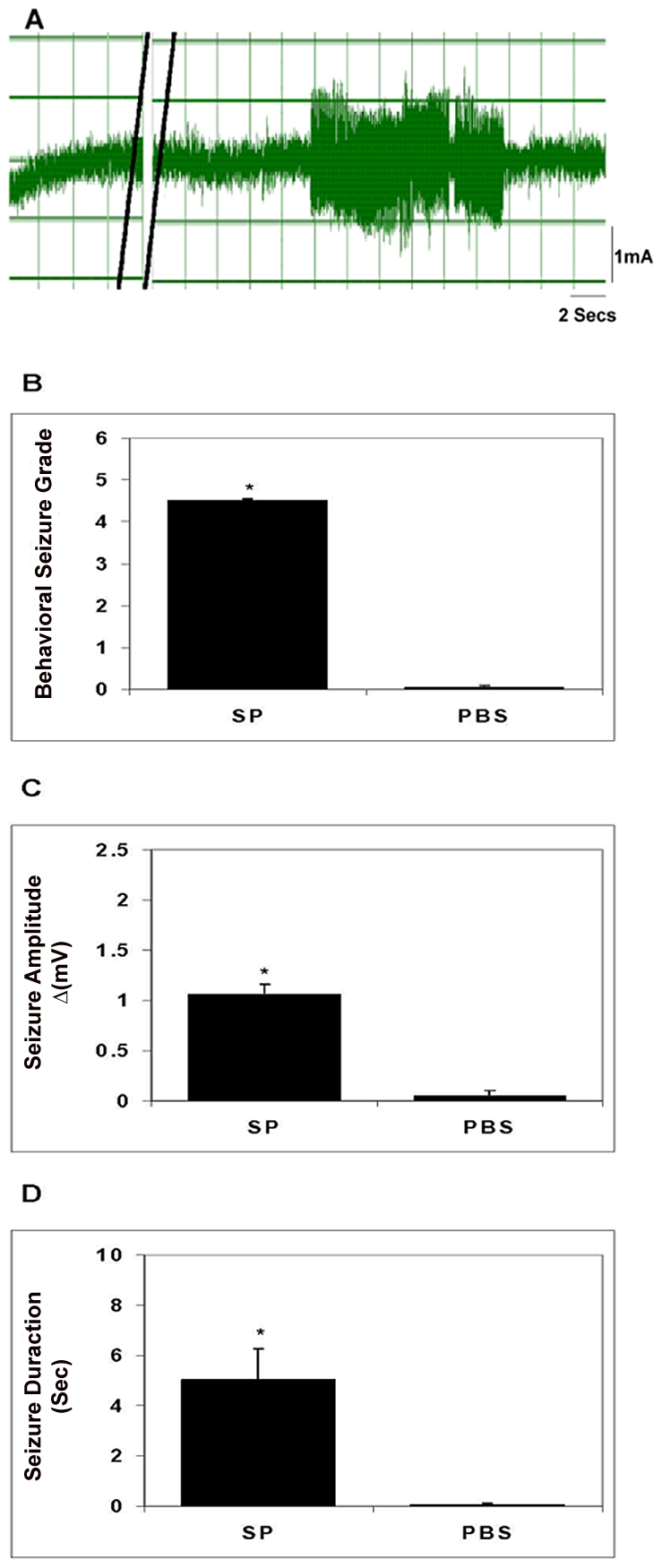
Seizure activity in rats following intrahippocampal injection of SP. A representative EEG recording before and after injection of SP into the hippocampus of a rat is shown in panel A; double black lines indicate period of SP injection. The behavioral seizure grade (B), seizure amplitude (C), and seizure duration (D) following intrahippocampal injection of SP or vehicle (PBS) in rats (n = 4–6) are depicted graphically (results shown are mean ± SEM; *, p≤0.001, Mann-Whitney). Results are pooled data from two independent experiments.

To determine if SP alone is capable of inducing seizures, we injected SP directly into the hippocampus of rats and examined them for seizure activity using direct observation and EEG monitoring (Figure 2). Injection of SP (10 nanomoles) resulted in severe behavioral seizures (Racine grade 4.5±0) and increased electrical activity on EEG (1.06±0.1 mV), which lasted 5.0±1.23 sec. In contrast, injection of PBS elicited essentially no seizure activity assessed by these parameters (p≤0.001, Mann-Whitney test, all comparisons). Thus, SP alone was epileptogenic in rats.

### Epileptogenic activity within early stage cysticercal granuloma extracts correlates with SP peptide levels and is blocked by pre-treatment with SP receptor antagonist (SRra)

**Figure 3:**
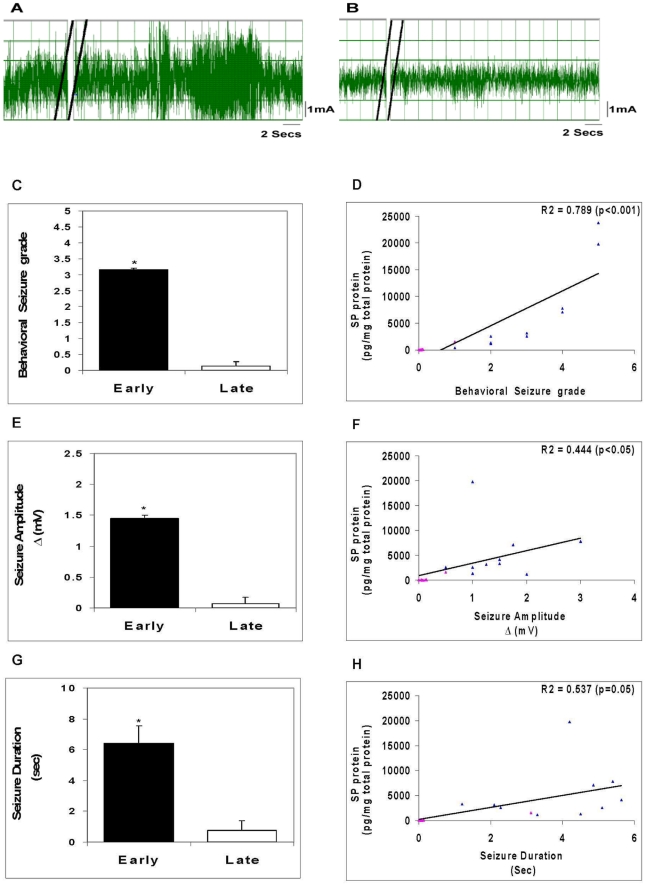
Seizure activity in rats following intrahippocampal injection of granuloma extracts. Representative EEG recordings after injection of an early granuloma extract (A) or a late granuloma extract (B) into the hippocampus of rat are shown; double black lines indicate period of extract injection. The behavioral seizure grade (C), seizure amplitude (E), and seizure duration (G) in rats following intrahippocampal injection of early or late granuloma extracts (n = 7–12) are depicted graphically (*, p≤0.001, Mann-Whitney). Correlation between levels of SP peptide within the granuloma extracts and behavioral seizure grade (D), seizure amplitude (F), and seizure duration (H) along with R^2^ and p values are shown (blue triangles represent each of the early stage granuloma extracts and pink triangles represent each of the late stage granuloma extracts). Results are pooled data from three independent experiments.

We previously demonstrated in the *T. crassiceps* murine model of cysticercosis that early and late stage granulomas form around the dead or dying parasites and can be isolated from peritoneal cavity [20], [21]. Early stage granulomas contained parasite remnants and produced Th1 cytokines and SP peptide, while late stage granulomas did not contain parasite remnants or SP and produced IL-4 in addition to Th1 cytokines [20], [21]. Injection of extracts of early, but not late stage granulomas into the hippocampus of rats induces seizure activity [22]. Since the ability of early granuloma extracts to induce seizures could not be attributed to the distinct cytokine profiles within the two stages [23], we explored the hypothesis that SP was responsible for seizures in this model of NCC, by examining the ability of extracts of early vs. late granulomas to induce seizures and determining whether or not a correlation existed between seizure activity and SP peptide level (Figure 3). Seizure activity in rats was induced in all cases following intrahippocampal injection of extracts of early stage granulomas (12 of 12). In contrast, seizure activity was induced in only 1 of 7 extracts of late granulomas (p<.005, Fishers exact test). In addition to the frequency of seizure induction, the severity of seizure activity was greater in rats receiving early vs. late granuloma extracts as determined by behavioral seizure grade (3.17±0 vs. 0.14±0.13; p≤0.001, Mann-Whitney test; Figure 3C), amplitude of EEG electrical activity (1.45±0 vs. 0.07±0.1 mV; p≤0.001, Mann-Whitney test; Figure 3E) and total duration of seizure activity (6.43±1.11 sec vs. 0.74±0.65 sec; p≤0.01, Mann-Whitney test; Figure 3G).

To determine the relationship, if any, between seizure activity and levels of SP peptide within extracts, we measured SP peptide in the extracts and correlated this with the intensity of seizure responses. There was a significantly positive correlation between SP levels and the behavioral seizure grade (R^2^ = 0.789, p<0.001, Pearson Correlation Coefficient; Figure 3D), the amplitude of seizure activity (R^2^ = 0.444, p<0.05, Pearson Correlation Coefficient; Figure 3F) and duration of seizure activity (R^2^ = 0.537, p≤0.05, Pearson Correlation Coefficient; Figure 3H). These findings add additional support to the hypothesis that SP contributes to the ability of early granuloma extracts to cause seizures.

### Effect of SP receptor antagonist (SPra) pretreatment on seizure responses induced by early granuloma extracts

**Figure 4:**
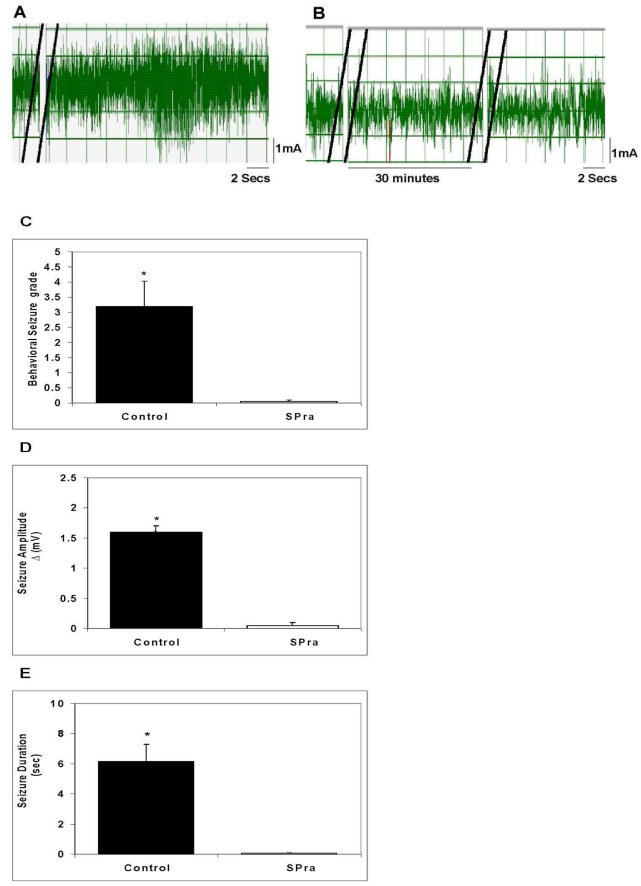
Effect of SP receptor antagonist (SPra) pre-treatment on granuloma extract-induced seizure activity in rats. Representative EEG recordings in rats after intrahippocampal injection of an early granuloma extract pretreated without (A) or with SPra (B) are shown; double black lines indicate the period of extract injection (A and B) or SP receptor antagonist injection (B only). The behavioral seizure grade (C), seizure amplitude (D), and seizure duration (E) in rats following intrahippocampal injection of early granuloma extracts pre-treated without (Control) or with SPra (SPra; n = 6 each) are depicted graphically (*, p≤0.001, Mann-Whitney). Results are pooled data from two independent experiments.

SP binds to its cognate receptor, NK1R, to mediate its effects. To support the contention that SP within early granulomas mediated the seizures, we examined the effects of pre-treatment with aprepitant, a SP receptor (NK1R) antagonist, on seizures induced by early stage granuloma extracts (Figure 4). Injection of aprepitant (1 µg) 30 min before injection of early stage granuloma extracts resulted in complete abrogation of EEG seizure activity (p≤0.001, Mann-Whitney test, all comparisons).

### Effect of SP receptor or NK1R deletion on seizure responses induced by early granuloma extracts

**Figure 5:**
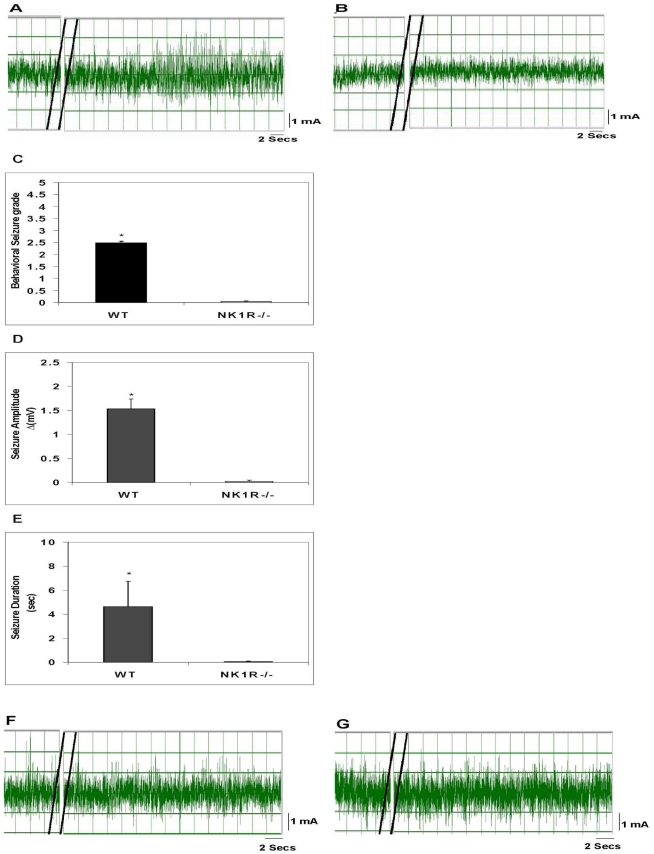
Effect of deletion of the NK1R gene or the SP gene in mice on granuloma extract-induced seizure activity. A representative EEG tracing after injection of an early granuloma extract obtained from a wild type mouse into the hippocampus of a wild type mouse (A) or NK1^−/−^ (B) mouse is shown. The behavioral seizure grade (C), seizure amplitude (D), and seizure duration (E) in wild type or NK1^−/−^ mice following intrahippocampal injection of extracts of early granulomas obtained from wild type mice (n = 4 each) are depicted graphically (*, p≤0.001, Mann-Whitney). EEG recordings shown (F and G) are representative of results obtained after intrahippocampal injection into a wild type mouse of an extract from an early granuloma (F) or late granuloma (G) obtained from an SPP^−/−^ mouse. Results are pooled data from two independent experiments.

To further support the above observation that SP was important for induction of seizure activity, experiments were performed using transgenic mice devoid of NK1R (NK1R^−/−^). We thus examined the effect of NK1R deletion on seizure activity induced by extracts of early granulomas (Figure 5). Similar to the results in rats, extracts of early granulomas obtained from wild type mice induced seizures when injected into the hippocampus of mice generating seizures with behavioral seizure grades of 2.5±0, electrical amplitude of 1.54±0.2 mV, and total duration of 4.63±2.13 sec. In contrast, no seizure activity was observed when extracts were injected into NK1R^−/−^ mice (p≤0.05, Mann-Whitney test, all comparisons).

### Effect of SP deletion on seizure responses induced by early granuloma extracts

To further establish that SP is the tachykinin within extracts of early granulomas that binds to NK1R within the brains of rodents to induce seizures, we examined whether early granulomas obtained from SP precursor knockout (SPP^−/−^) mice were capable of inducing seizures when injected into the hippocampus of wild type mice (Figure 5). Twelve granulomas from infected SPP^−/−^ mice were examined—7 early stage and 5 late stage. Neither early nor late granuloma extracts from these mice induced seizure activity.

## Discussion

We demonstrated that SP-producing cells are present in the brain of NCC patients and are localized specifically to areas of inflammation adjacent to the degenerating worms. Also, severe seizures occurred in rats after intrahippocampal injection of SP alone, as well as after injection of SP-containing extracts of granulomas obtained from *T. crassiceps*-infected wild type (WT) mice. In addition, seizure activity correlated with SP levels within extracts and was prevented by intrahippocampal pre-injection of aprepitant, a SP receptor antagonist. Furthermore, studies using mice deficient in SP precursor or SP receptor (NK1R) demonstrated that SP precursor was required to generate epileptogenic granuloma extracts, and that the SP receptor (NK1R) was required to respond to these extracts with seizure activity, respectively. Taken together these findings strongly suggest that SP produced within cysticercal granuloma causes seizures in rodent models of NCC and together with the SP localization findings in NCC patients suggest that seizures can be prevented and/or treated in human NCC with SP receptor antagonists.

SP peptide is encoded by the preprotachykin A gene. The protein product of this gene, preprotachykinin A, is cleaved to form two active neuropeptides, SP and neurokinin A. The SP precursor (preprotachykinin) knockout mice used in our studies are deficient in both SP and neurokinin A. Consequently, our results in SPP-knockout mice can be attributed to a deficiency in either SP or neurokinin A. The possibility that neurokinin A, and not SP, is mediating seizures in NCC is unlikely for several reasons. First, the SP antibody used in the brain immunohistochemistry studies was specific for SP and did not cross-react with neurokinin A. Also, while neurokinin A binds to NK1R, it binds with much lower affinity than SP; while, the opposite is true for NK2R, NK2R binds neurokinin A with much greater affinity than SP. Our results demonstrated a complete absence of seizures in NK1R-knockout mice, which express NK2R. If neurokinin A was the mediator of seizures in our models of NCC, we would have anticipated persistent epileptogenic activity within granuloma extracts in these mice.

The mouse model of cysticercosis due to *Taenia crassiceps* used in these studies has previously been exploited by us and others as a model to study the molecular pathogenesis of NCC [24]–[27]. Similar to *T. solium* infection in humans, live *T. crassiceps* cysts in mice cause little or no inflammation, while dead or dying parasites initiate a granulomatous reaction. The inflammatory response that occurs around dead or dying *T. crassiceps* cysts can be separated into early stage and late stage granulomas based on the extent of destruction of the parasite, their cytokine profile, and their pattern of SP peptide production [20], [21]; early stage granulomas contain parasite remnants, produce Th1 cytokines, and SP peptide while late stage granulomas contain neither parasite remnants nor SP and produce IL-4 in addition to Th1 cytokines [20], [21]. Cytokines such as TNF-α, IL-1β and IL-6 [28], [29] have been shown to induce seizures, However, in previous studies we were unable to attribute the epileptogenicity of early granuloma extracts to their distinct cytokine profile [20], [23]. Our current findings provide definitive support to the conclusion that it is not the repertoire of cytokines present within early granuloma that induces seizures, rather, the presence of SP peptide.

In addition to demonstrating that SP within cysticercal granulomas causes seizures in a rodent model of NCC, this is the first demonstration that SP alone causes seizures. SP previously had been shown to evoke epileptiform responses in neurons [30]. Also, mice with disruption of the preprotachykinin A gene showed a reduction in the duration and severity of seizures induced by intrahippocampal administration of kainic acid or pentylenetetrazol [19]. In addition, Liu et al demonstrated that while administration of SP (10 pmol) alone into the hippocampus of rats had no effect, SP administration combined with stimulation of the perforant path within the hippocampus resulted in severe self-sustaining status epilepticus (SSSE) and generated a pattern of acute hippocampal damage resembling that seen in status epilepticus in patients [18]. Our findings establishing that SP alone causes seizures raises the possibility that it contributes to seizures seen in other infectious and/or inflammatory diseases involving the brain.

These studies have potential implications for treatment and prevention of seizures in the setting of NCC especially seizures induced by antihelmintic treatment of NCC patients with viable cysts. Current management options for patients with moderate infections and viable cysts include antihelmintic treatment along with administration of corticosteroids to reduce inflammation and the predisposition to seizures initiated by dying parasites. Antiepileptic drugs are used to treat NCC patients with spontaneously-occurring seizures [31], [32] and to prevent future attacks; many patients must resign themselves to taking these drugs for the rest of their lives [32]. However, both corticosteroids and antiepileptic drugs can have severe side effects. Our finding that SP causes seizures in NCC precipitated by the granulomatous response to dead or dying cysts suggest the possibility of using SP receptor antagonist for seizure prophylaxis during periods of antihelmintic treatment and, perhaps, as an adjuvant or replacement for anti-epileptic drugs for the treatment and/or prevention of spontaneous seizures in NCC.

In addition to being induced by early granuloma surrounding dead or dying cysts, seizures in NCC have been associated with calcified lesions, which presumably represent end-stage granuloma or scaring. Of note, calcified lesions with surrounding edema, in particular, have been associated with episodic seizure activity [33]–[35]. Based on our findings, it is tempting to speculate that SP within the inflammatory response leading to edema around calcified cysts causes seizures in this setting as well. However, support for this hypothesis awaits further studies.

## Materials and Methods

### Brain immunohistochemistry

Human studies were approved the Institutional Review Board of Baylor College of Medicine. Human subjects underwent neurosurgical procedures as part of their clinical standard of care and they provided written informed consent to their treating physicians prior to surgery. After diagnostic histopathology evaluation was done, remaining tissues were archived and would ultimately be discarded. Human Institutional Review Board of Baylor College of Medicine approval was obtained to use de-identified paraffin sections from this archival material. Human brain tissue sections derived from 5 patients who were diagnosed with NCC associated seizures were used to probe for SP peptide by immunohistochemistry. Human brain tissue from 2 normal individuals who had died due to other brain-related causes was used as control. Immunoperoxidase staining was performed on 5 µm thick paraformaldehyde-fixed human brain sections by using the avidin-biotin method, an automated immunostainer (Biogenex), and polyclonal rabbit antibody to SP (1∶2500; Chemicon, Temecula, Calif.) or control rabbit serum. Slides were scored on the basis of positive staining demonstrated within the cytoplasm of cells at a level above the level of the nonspecific signal in tissue cells. Five-to-ten high-power fields (1000×) of each slide were scored by an experienced microscopist blinded to the study design. The slides were graded on a scale of 0 to 4+ as follows, 0–0.5+- no positive cells, faint diffuse staining; 1+, 1–10% of cells positive, 2+, >10–20% of the cells positive; 3+, >20–30% of the cells positive; and 4+, >30% of the cells positive.

### Animal studies

All studies with animals were approved by the Institutional Animal Care and Use Committee of Baylor College of Medicine. Use of all animals involved in this project were carried out according to the provisions of the Animal Welfare Act, PHS Animal Welfare Policy, the principals of the NIH Guide for the Care and Use of Laboratory Animals, and the policies and procedures of Baylor College of Medicine. All surgery was performed under DEA Schedule III anesthesia, and all efforts were made to minimize suffering.

### Mice

Six-week-old WT C57/BL6 mice were purchased from Jackson Laboratories. Tachykinin 1 or SP precursor knockout mice (SPP^−/−^) mice were generated, as described [36]. Briefly, a targeting vector containing neomycin resistance and thymidine kinase genes was used to disrupt the Tac1 gene, replacing the SP exon 3 with neo and deleting the neurokinin A exon 6. Chimeric animals were backcrossed into the C57BL/6J for 15 generations. SPP^−/−^ mice are viable, fertile, normal in size and do not display any gross physical or behavioral abnormalities. Nociceptive pain responses and neurogenic inflammation are absent. SPP^−/−^ male and female mice were obtained from Dr. Julio Fontan, Southwestern Medical Center, Dallas, Texas and bred within the BCM animal facility. NK-1R^−/−^ were generated as described [37]. Briefly, a targeting vector was generated in which exon 1 of the NK-1R gene was partially deleted, including the initiating methionine codon, and replaced with a cassette encoding *lacZ* and neomycin resistance. Chimeric animals were backcrossed into the C57BL/6J for at least 15 generations. The homozygous NK1R^−/−^ mice expressed no detectable NK1 receptor peptide, were grossly normal developmentally, were fertile, and appeared to be healthy under barrier isolation conditions. NK1R^−/−^ mice were originally obtained from the laboratory of Dr. Joel Weinstock, Tufts University School of Medicine, Boston, Massachusetts.

### Rats

Adult Sprague Dawley rats (125–175 grams) were purchased from Harlan Laboratories (Houston, Texas).

### Murine cysticercosis model

Female wild type and SPP^−/−^ mice (15–20 mice per group) were infected by intraperitoneal inoculation with 10 cysts of the ORF strain of T. crassiceps, as previously described [21], [38]. All infected animals were housed in a BSL2 facility at Baylor College of Medicine. Three months following infection, the mice were euthanatized by cervical dislocation under anesthesia. Granulomas associated with parasites were identified visually, removed from the peritoneal cavity, portioned into 3 parts and used for different experiments as follows. One part of the granulomas was fixed in 4% paraformaldehyde to be utilized for histological staging, the second part of the granuloma was frozen in liquid nitrogen to be used for quantitation of SP peptide. The remainder was washed once, followed by homogenization in phosphate buffered saline to generate extracts for study of epileptogenic responses.

### Granuloma staging

The portion of each granuloma fixed with 4% paraformaldehyde was paraffin-embedded and cut into 5-µm sections, which were then stained with Giemsa and examined microscopically. We separated the granulomas into early and late stages based on the histological appearance of the degenerating parasite according to previously described methods [20].

### Extraction and quantitation of Substance P

Early and late stage granulomas were homogenized in 1% trifluoroacetic acid (TFA) (1 ml/gram of tissue) and centrifuged at 17,000 g for 15 minutes at 2–8°C. A Sep-Pak C18 Cartridge (Waters, Associates, Milford, MA) was prewetted with 100% acetonitrile followed by 1% TFA in water. The supernatant was then passed through the cartridge, followed by a wash with 10–20 ml of 1% TFA. Protein was then eluted with 3 ml of a 60∶40 solution of acetonitrile∶1% TFA and dried using a centrifugal concentrator under vacuum. The dried samples were then reconstituted in assay buffer containing protease inhibitor, aprotinin (500 KIU/ml, Sigma) and quantitated using ELISA kit from R&D Biosystems. Results are expressed as picograms of SP in 1 mg of total protein. Total protein was quantitated using the Bradford method (cat no. 500-0006, Bio-Rad, Hercules, CA).

### Generation of granuloma extracts for seizure experiments

To prepare extracts for seizure experiments, each granuloma was homogenized in phosphate buffer saline. The homogenate was centrifuged at 10,000 rpm for 10 minutes and the supernatant used for seizure experiments as underlined below. Total protein in the supernatant was quantitated using the Bradford method (cat no. 500-0006, Bio-Rad, Hercules, CA).

### Rat and mouse seizure models

#### Examination of epileptogenic responses in rats

The rat seizure model was performed, as previously described [22]. Briefly, adult Sprague Dawley rats were anesthetized with ketamine/acepromazine/xylazine (25/5/0.8 mg/kg) and placed in a stereotaxic frame. A burr hole was drilled in the location above the hippocampus area and a small tube and wire was lowered into the hippocampus and cemented in place. A PE 10 tubing that was attached to a 10 µl Hamilton syringe was attached to the small tube in the brain. EEG activity of anesthetized rats and behavioral seizures of conscious rats were observed after injection of 10 microliters of SP (10 nM) or granuloma extract supernatant containing 25 µg of total protein into the brain. For recording of EEG activity, a male electrical connector was soldered to the wire, attached to an amplifier and then to a chart recorder. Results of EEG recordings are expressed as seizure duration (duration time in seconds) and seizure amplitude (Δ amplitude of seizure activity calculated by subtracting baseline EEG amplitude from the maximal seizure amplitude activity expressed in milli volts). For observation of behavioral seizures, the animal was allowed to recover fully from the anesthetic before injection of test sample. Behavioral changes were observed and carefully described for up to 6 hours after the injection. Behavioral seizures were scored according to a previously defined scale of Racine et al [39]. Briefly, this rating scale classifies mouth and face twitches and clonic head movements as stages 1 and 2 respectively, the appearance of contralateral forelimb clonus is designated as stage 3. The progression to stage 4 is characterized by bilateral forelimb clonus that is now associated with rearing. Stage 5 seizures involve bilateral forelimb clonus with rearing and falling. Where indicated, 1 microgram of SP receptor antagonist (Emend, Merck, Whitehouse, NJ) was injected 15–20 minutes prior to intrahippocampal injection of test sample. Controls included injection of rats with vehicle or SP receptor antagonist alone.

#### Examination of epileptogenic responses in mice

Briefly, adult mice were anesthetized with ketamine/xylazine (80/16 mg/kg) and placed in a stereotaxic frame. The mice were injected intrahippocampally with granuloma extracts as mentioned above. Three microlitres of the granuloma extracts containing 7–10 ug of protein were used for intrahippocampal injection in anaesthetized adult WT mice and anaesthetized NK1R^−/−^ C57BL/6 mice. The mice were then monitored for EEG seizure activity and behavioral seizure activity as described above.

### Identification of accuracy of injection site

Twenty-four hours after intrahippocampal injection, animals were anesthetized with urethane (1.2 g/kg) and perfused through the heart with 4% paraformaldehyde. The brain tissues were equilibrated in 30% sucrose and 35 µm horizontal sections were Nissl stained and examined for the needle track/injection site.

### Statistical analyses

Statistical differences were determined using the Mann-Whitney test, Student's t-test or Pearson's correlation test, as indicated.
